# Decoding Tumor–Immune Interactions in Hepatocellular Carcinoma Through Network-Centered Identification of CXCR2

**DOI:** 10.3390/ijms27167501

**Published:** 2026-08-21

**Authors:** Saleh A. Almatroodi, Tarique Sarwar, Arshad Husain Rahmani

**Affiliations:** Department of Medical Laboratories, College of Applied Medical Sciences, Qassim University, Buraydah 51452, Saudi Arabiat.sarwar@qu.edu.sa (T.S.)

**Keywords:** prognosis, WGCNA, cancer, hepatocellular carcinoma, PPIN, *CXCR2*

## Abstract

Hepatocellular carcinoma (HCC) is one of the most prevalent cancers worldwide and exhibits considerable biological heterogeneity in both molecular and clinical characteristics. The diverse molecular alterations and clinical manifestations of HCC indicate substantial heterogeneity across patient subgroups. This study aimed to identify novel therapeutic targets and predictive biomarkers associated with HCC using an integrative bioinformatics approach. High-throughput genomic datasets were obtained from the UCSC Xena browser to retrieve mRNA HTSeq-count data from the TCGA-HCC cohort. Gene co-expression network (GCN), protein–protein interaction network (PPIN), and enrichment analyses were performed to identify key dysregulated genes and their biological significance. Integrated network analyses identified three dysregulated hub genes, namely CXCR2, TLR2, and TLR4. Genomic alterations in these genes were further evaluated across tumor samples in the TCGA-HCC cohort. Kaplan–Meier (KM) survival analysis demonstrated that lower CXCR2 mRNA expression was significantly associated with poorer overall survival (OS) and recurrence-free survival (RFS). Furthermore, TIMER and UALCAN analyses revealed significant associations between CXCR2 expression and tumor purity, as well as immune cell infiltration levels, including T cells, macrophages, dendritic cells (DCs), and neutrophils. These findings suggest that CXCR2 is significantly associated with the immune microenvironment of HCC and represents a potential prognostic biomarker whose biological role warrants further mechanistic investigation.

## 1. Introduction

The third leading cause of cancer-related death worldwide is liver cancer, which is also the sixth most common disease overall. According to the 2020 Global Cancer Statistics report, there have been fatalities globally, as well as bad prognosis for liver cancer, which is highlighted by new cases [1]. It is predicted that by 2040, there will be an increase in liver cancer incidence. The most frequent malignancy is hepatocellular carcinoma (HCC) [2]. HCC, a kind of liver cancer, is the second most common cause of cancer-related deaths and is becoming more prevalent everywhere [3]. Hepatocytes, the primary class of liver cells, are where it primarily develops. HCC can develop in people without underlying liver disease; however, it most frequently does so in the presence of cirrhosis and chronic liver disease [4]. Several factors increase the risk of developing HCC, including chronic hepatitis B or C infection [5], cirrhosis (usually due to alcohol abuse or non-alcoholic fatty liver disease (NAFLD)), exposure to aflatoxins (a type of toxin produced by certain molds on peanuts and grains), and certain genetic conditions like hemochromatosis. In the early stages, HCC may not cause noticeable symptoms [6].

HCC etiology is a multifaceted, complex process that includes several genetic, environmental, and molecular variables [7]. Chronic liver disease normally develops over a long period of time, and the process can be broken down into several crucial components. These diseases can cause inflammation and liver cell damage, which pave the way for the growth of HCC. Chronic liver inflammation and injury may ultimately lead to the development of scar tissue (fibrosis) [7]. In addition to fibrosis and chronic inflammation, liver cells undergo biochemical and genetic alterations. Normal liver cells can develop into malignant cells due to mutations and changes in important regulatory genes. For instance, HCC frequently presents mutations in the TP53 tumor suppressor gene [8]. The growth of cancer cells can be aided by the abnormal activation of signaling pathways involved in cell survival, proliferation, and angiogenesis. The Wnt/-catenin pathway, the PI3K/AKT/mTOR pathway, and the Ras-MAPK pathway are important pathways connected to the development of HCC [9]. As cancer progresses, common symptoms can include abdominal pain or discomfort, unexplained weight loss, jaundice (yellowing of the skin and eyes), fatigue, and abdominal swelling. HCC treatment is still difficult and heavily dependent on early detection. Early identification of HCC is essential for enhancing patient survival and treatment success. Whether various biomarkers can identify HCC in its early phases has been investigated. One of the most extensively researched biomarkers for HCC is alpha-fetoprotein (AFP) [10]. Although AFP levels can be raised in HCC, they can also be upregulated in other liver diseases. Another biomarker that may be increased in HCC is des-gamma-carboxyprothrombin (DCP). When AFP levels are only mildly increased, it is especially helpful. DCP tests can be used in addition to AFP measures to diagnose HCC [11]. Similarly, other biomarkers include AFP-L3, PIVKA-II, *GPC3*, and *OPN* [12,13]. Targeted therapy has made significant strides in recent years, yet the results of treating cancer remain unsatisfactory. Therefore, it is critical to comprehend the molecular pathways underlying the development of HCC and to find novel molecular targets and new prognostic indicators [14]. Additionally, this will offer profound insight into every step of HCC diagnosis and therapy. Currently, in silico methods are frequently utilized to find important regulatory genes and to ascertain their structural and relational roles in disease [15,16,17]. This strategy provides the best means to search massive gene expression profiles of healthy and clinical populations in order to comprehend the genetic pathways influencing the onset and course of various diseases. To identify differences in gene expression between cancerous and non-cancerous tissues, evaluate differentially expressed genes (DEGs), and identify the pathways that contribute to carcinogenesis and the development of cancer, high-throughput microarray technology and bioinformatics analysis are now frequently used in different cancers [18]. Cancer-associated gene expression profiles have revealed new biomarkers and treatment targets that have produced consistent results in clinical research [19]. Recent research reported four genes, *APLN*, *PCK1*, *ALDH2*, and *E2F1*, that were strongly related to HCC [2]. Similarly, a recent finding provided justification for considering hypericin as a potential anticancer drug for CCNB1-mediated HCC [20]. In the present work, we retrieved messenger RNA (mRNA) count data from the UCSC Xena browser corresponding to the Cancer Genome Atlas (TCGA)-HCC cohort patient samples. Following differential expression analysis (DEA), all the DEGs were subjected to robust and significant trait-linked hub module(s) identification via weighted gene co-expression network analysis (WGCNA). Protein–protein interaction network (PPIN) and enrichment analyses of WGCNA hub module(s) DEGs were carried out thereafter. Later, the DEGs overlapping between the top-scoring PPIN cluster and significant pathway/gene ontology (GO) terms data were considered as hub DEG(s). Mutational and overall survival (OS) analyses of hub DEG(s) were performed, followed by external validation using databases such as TIMER and UALCAN.

Our work’s novelty lies in the application of the selection of patient samples and the stringent application of pre-processing and batch correction algorithms, unlike already published work in the same field. Trait-linked hub module(s) selection via WGCNA, followed by PPIN and enrichment analyses, assist in the elucidation of a handful of significant predictive biomarkers. Prognostic and mutational analyses affirm these biomarkers’ vital role in HCC pathogenesis from a bioinformatics perspective. Lastly, external validation of prognostic biomarkers using TIMER and UALCAN unifies the molecular biology concepts, such as immune cell distribution in TME and promoter methylation status, with prognostic biomarkers. However, further in vitro and in vivo experimentation is greatly needed to validate these predictive biomarkers’ roles in early HCC diagnosis and personalized treatment.

## 2. Results

### 2.1. HCC-Associated RNA-Seq Data Extraction and DEA

**Figure 1:**
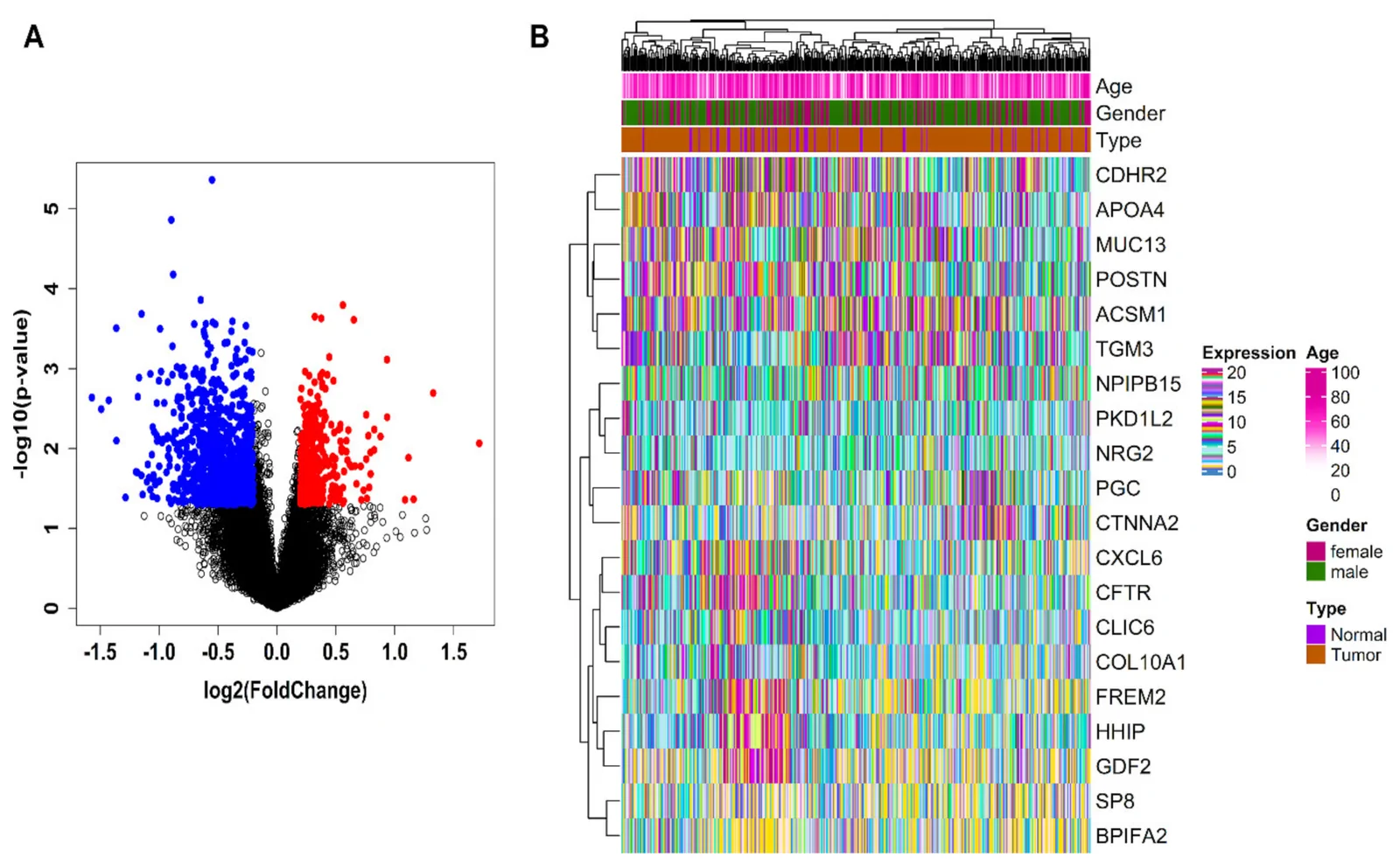
(**A**) Volcano plot indicating distribution of HCC-associated DEGs (red and blue dots indicate upregulation and downregulation) and non-significant genes (black dots), respectively. (**B**) Annotation heatmap representing expression distribution of top 20 HCC-associated DEGs (10 up and 10 downregulated) across tumor and healthy patient samples. Left and top sides of plot signify cluster dendrograms demonstrating Euclidean distance-based hierarchical clustering for columns and rows. Sample type and gender annotation bars are displayed at top of heatmap.

As per the above-mentioned inclusion criteria, we extracted the HCC cohort comprising 360 patient samples. Table S1 summarizes the clinical information related to the HCC cohort. In total, 1582 DEGs (1108 downregulated and 474 upregulated) were identified, corresponding to a *p*-value < 0.05 and $\left|{log}_{2}(fold\;change)\right|>0.2$. A volcano plot showing the significant (red and blue colored dots) and non-significant (black-colored points) genes is shown in Figure 1A. A heatmap plot showing the top 20 HCC-associated DEGs (10 up + 10 downregulated) across HCC and healthy patient samples is shown in Figure 1B. The most highly down- and upregulated DEGs were *FREM2* [${log}_{2}(fold\;change)=-1.57$] and *PGC* [${log}_{2}(fold\;change)=1.71$].

### 2.2. HCC-Associated WGCN Formation and Hub Module Selection

**Figure 2:**
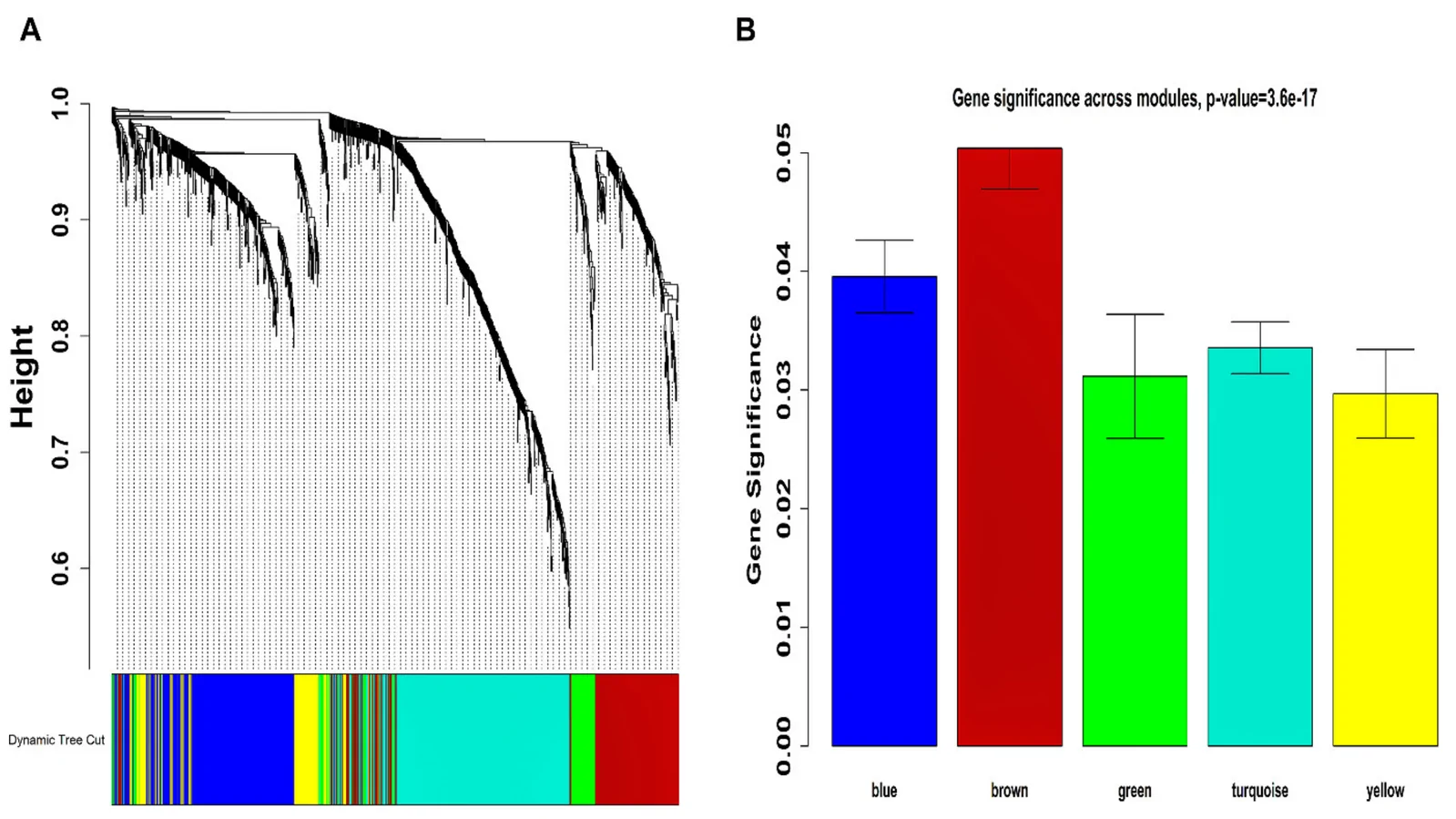

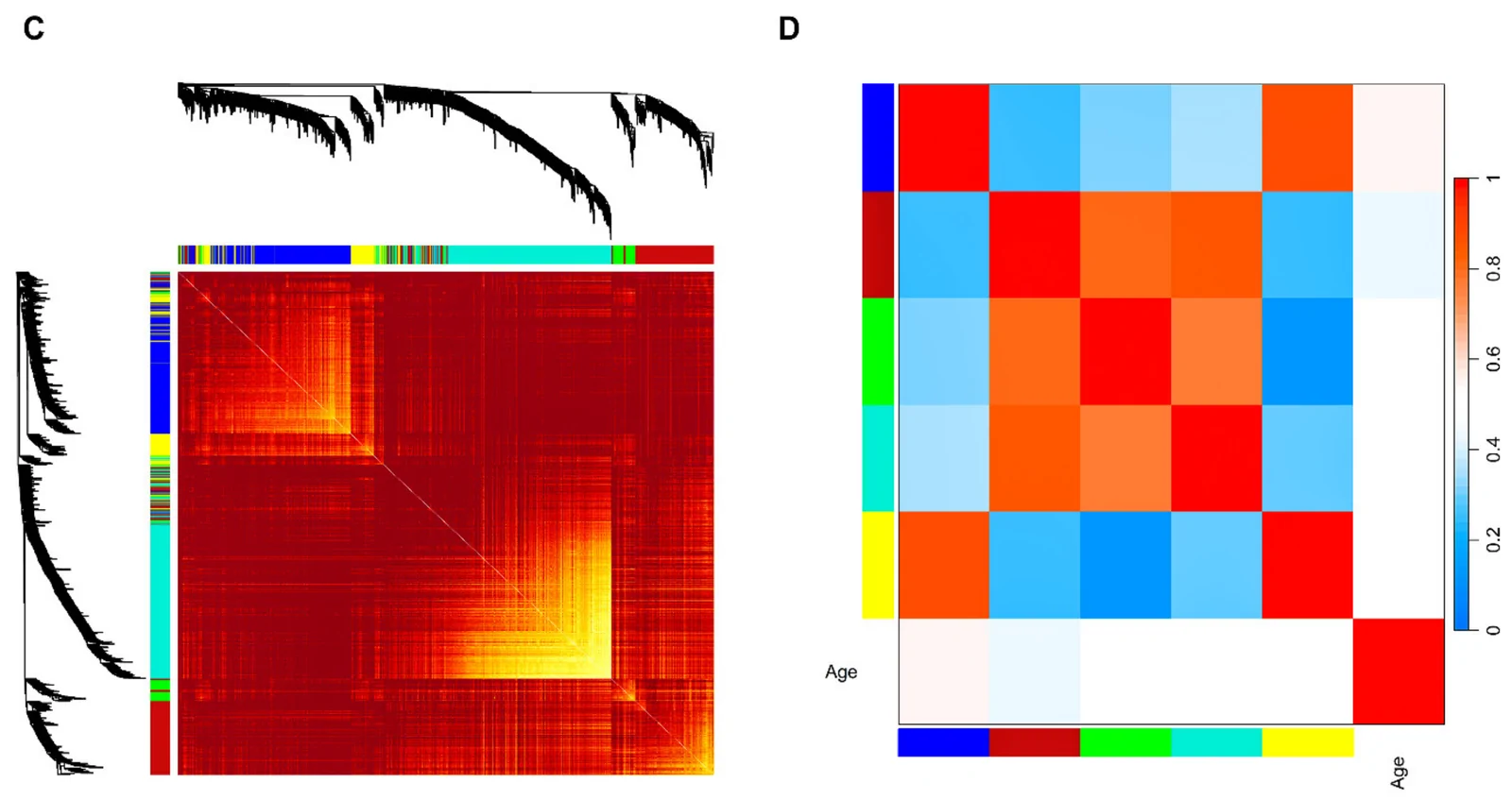
(**A**) The hierarchical clustering dendrogram illustrates the grouping of 1250 HCC-associated DEGs based on dissTOM, forming five color-coded communities (yellow, blue, turquoise, green, and brown), with module sizes ranging from 78 to 452 DEGs. (**B**) The barplot presents the distribution of GS along with error bars for the five modules. (**C**) The WGCN is depicted as a TOM plot, with DEGs organized in columns and rows and hierarchically clustered by cluster dendrograms. The varying shades of red within the matrix signify different levels of topological overlap among DEGs. Dark-colored blocks along the diagonal indicate module boundaries. (**D**) The eigengene adjacency heatmap showcases the interrelationships among the five co-expression modules. These values are based on linear regression coefficients of determination obtained from the module eigengene (ME) values in WGCNA. The color scale represents correlations, with red indicating values between 0.5 and 1 and blue indicating values between 0 and 0.5.

The expression data of 1250 non-noisy HCC-associated DEGs across 357 non-outlier samples were used to establish a WGCN. In accordance with scale-free topology (SFT), β=5 (corresponding to $R^{2}=0.84$) was used for the creation of WGCN. Figure S1A–D show plots for validating the β value in accordance with SFT criteria. Figure 2A shows five communities (i.e., blue: 316 DEGs; brown: 267 DEGs; green: 78 DEGs; turquoise: 452 DEGs; yellow: 137 DEGs) identified from the clustering (hierarchical) tree and dynamic tree cut (DTC) algorithm. Figure 2B shows a gene significance (GS) barplot (correlated with age) across all modules, where the brown module (module significance = 0.05) was the most highly correlated compared to the others. The gene co-expression network (GCN) plot in Figure 2C captures the topological overlap matrix (TOM) information for all module DEGs. The eigengene heatmap highlighting groups of highly correlated eigengenes is shown in Figure 2D as an eigengene adjacency heatmap. Tables S2–S4 summarize the module membership (MM) versus intramodular connectivity (k.in), MM versus GS, and GS versus k.in correlation values. Based on the trend observed, the turquoise module was selected as our hub module.

### 2.3. Functional Enrichment and PPIN Modular Analyses

**Figure 3:**
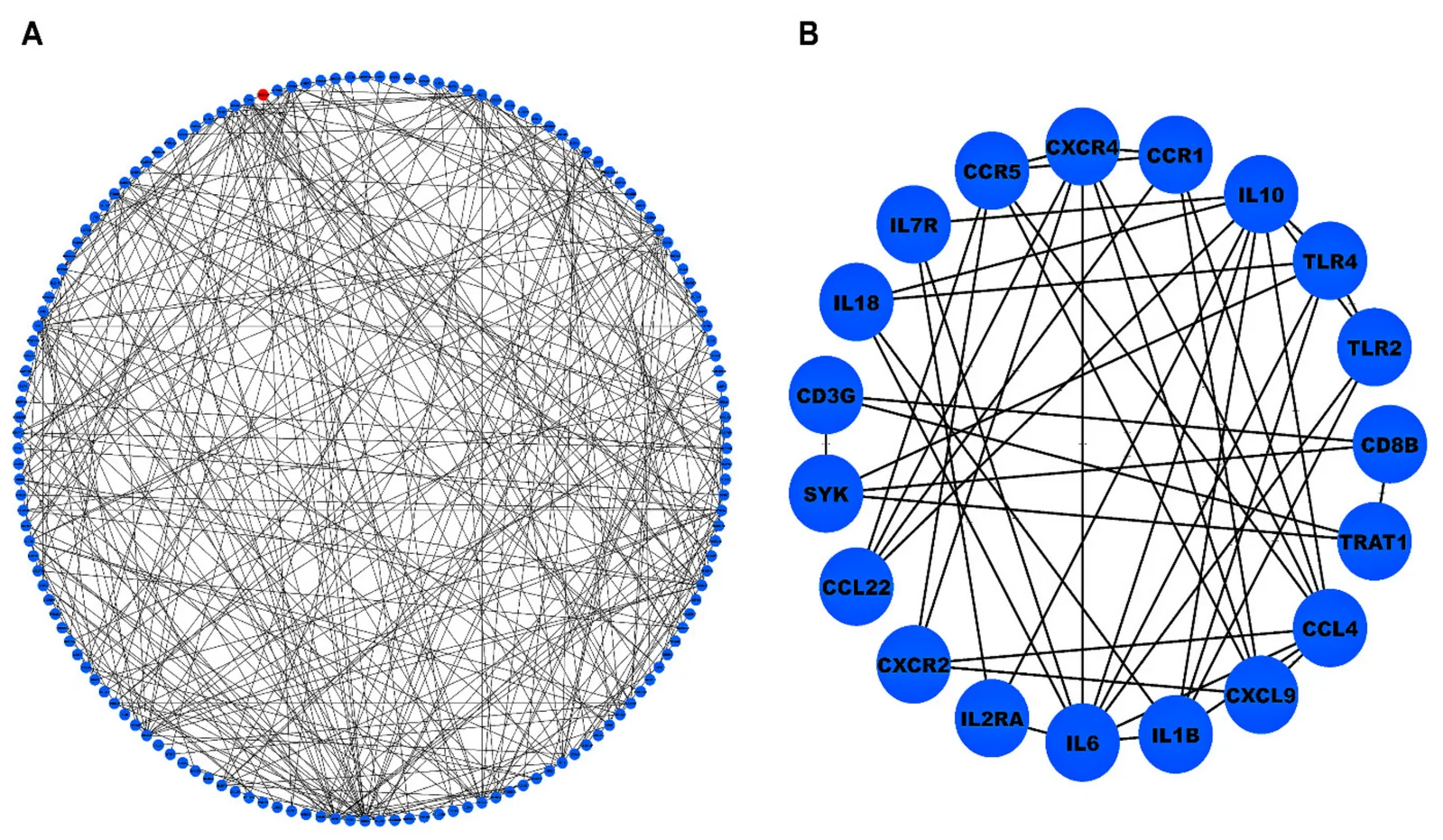
(**A**) Unweighted and undirected PPIN comprising 150 nodes and 316 edges corresponding to STRING interaction score > 0.9. (**B**) Top-scoring PPIN cluster comprising 19 nodes and 48 edges. Red-and blue-colored nodes signify up- and downregulated expression status of DEGs.

**Figure 4:**
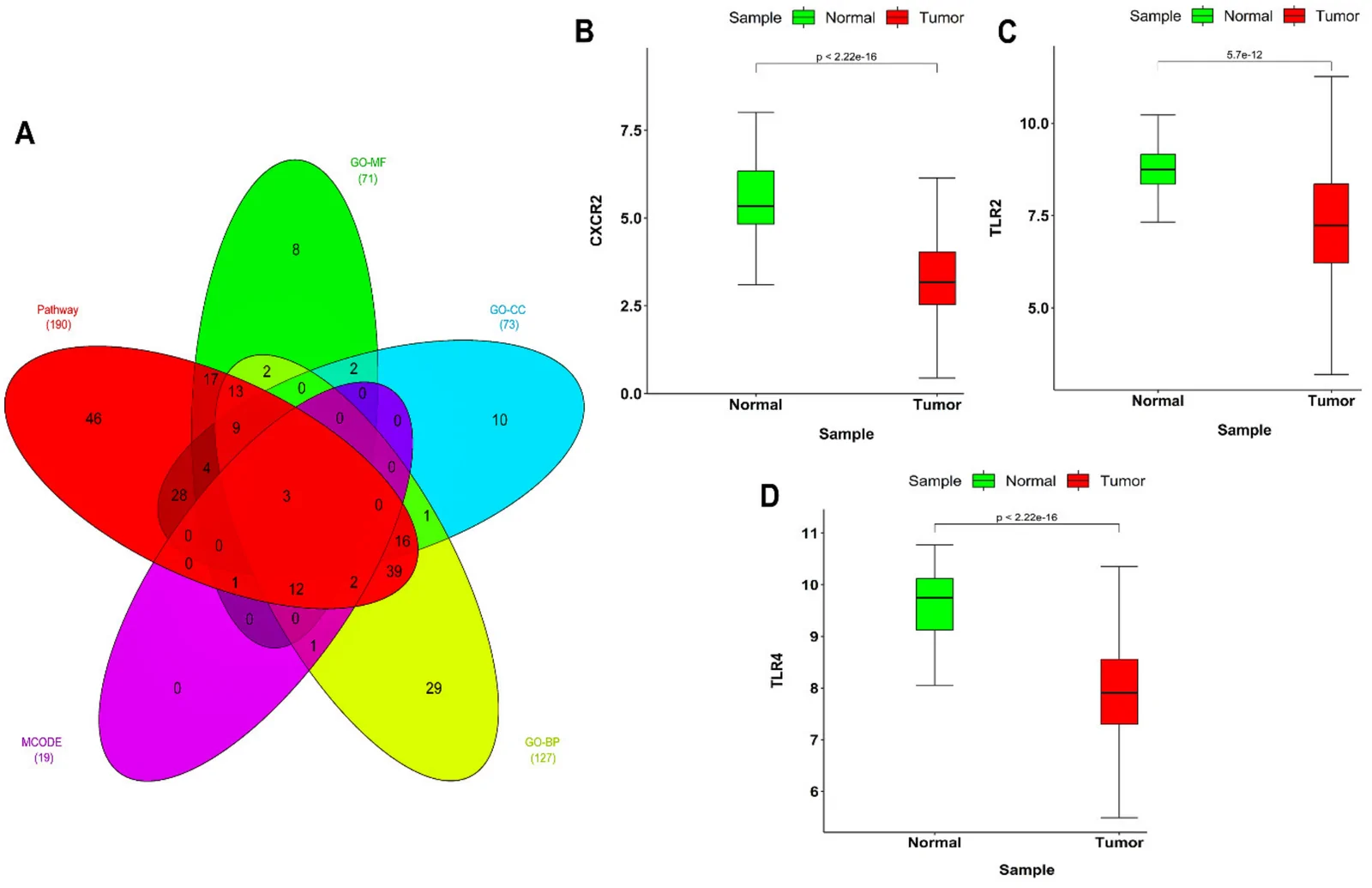
(**A**) Venn plot representing overlapping hub DEGs between top-scoring PPIN cluster, top 10 significant pathways, top 10 significant GO-BP, top 10 significant GO-MF, and top 10 significant GO-CC gene sets. Red-, green-, cyan-, yellow-, and magenta-colored areas correspond to pathway, GO-MF, GO-CC, GO-BP, and MCODE gene sets, respectively. Box-and-whisker plots showing mRNA expression distribution of (**B**) CXCR2, (**C**) TLR2, (**D**) TLR4. In comparison of TLR4 expression between normal and HCC tumor samples, normal samples are represented by green-colored boxes, while tumor samples are depicted in red. Top and bottom edges of boxes correspond to 75th and 25th percentiles of distribution, respectively. Horizontal lines within boxes represent median values, and minimum and maximum values are indicated at endpoints of axes.

Tables S5–S8 show a list of the top 10 significantly enriched pathways and GO terms corresponding to WGCN hub module DEGs. The most significant pathway, GO-molecular function (MF), GO-cellular compartment (CC), and GO-biological process (BP) terms were immune system (BH-*p* value $=4.01\times 10^{-41}$), major histocompatibility complex (MHC) protein binding (BH-*p* value $=1.75\times 10^{-5}$), secretory granule membrane (BH-*p* value $=2.20\times 10^{-20}$), and inflammatory response (BH-*p* value $=1.27\times 10^{-29}$). The unweighted and undirected PPIN, as shown in Figure 3A, comprises 150 nodes linked by 316 edges. Figure 3B shows the top-scoring PPIN cluster (MCODE score = 5.33), which comprises 19 nodes and 48 edges. Both *IL6* and *IL10* had the highest degrees (i.e., 9) in the top-scoring PPIN cluster. The Venn plot, as shown in Figure 4A, shows 3 hub DEGs (i.e., *CXCR2*, *TLR2*, *TLR4*) overlapping between the top-scoring PPIN cluster, top 10 significant pathways, top 10 significant GO-MF, top 10 significant GO-BP, and top 10 significant GO-CC gene sets. Figure 4B–D display box-and-whisker plots illustrating the relative expression distributions of *CXCR2*, *TLR2*, and *TLR4* in HCC patient samples as compared to those from healthy individuals. Notably, the mRNA expression levels of all key DEGs were significantly reduced in tumor samples compared to the samples from healthy individuals.

### 2.4. Validation of Hub DEGs via Gene Expression Omnibus (GEO)

We selected GSE84005, comprising 38 paired non-tumor and adjacent tumor tissue samples from HCC patients. Corresponding to the above-mentioned threshold, we obtained a total of 3263 DEGs, as shown in Table S9. All the hub DEGs (i.e., *CXCR2*, *TLR2*, *TLR4*) were present in the DEG list, hence confirming their validation in an external GEO cohort.

### 2.5. Mutational and OS Analyses

**Figure 5:**
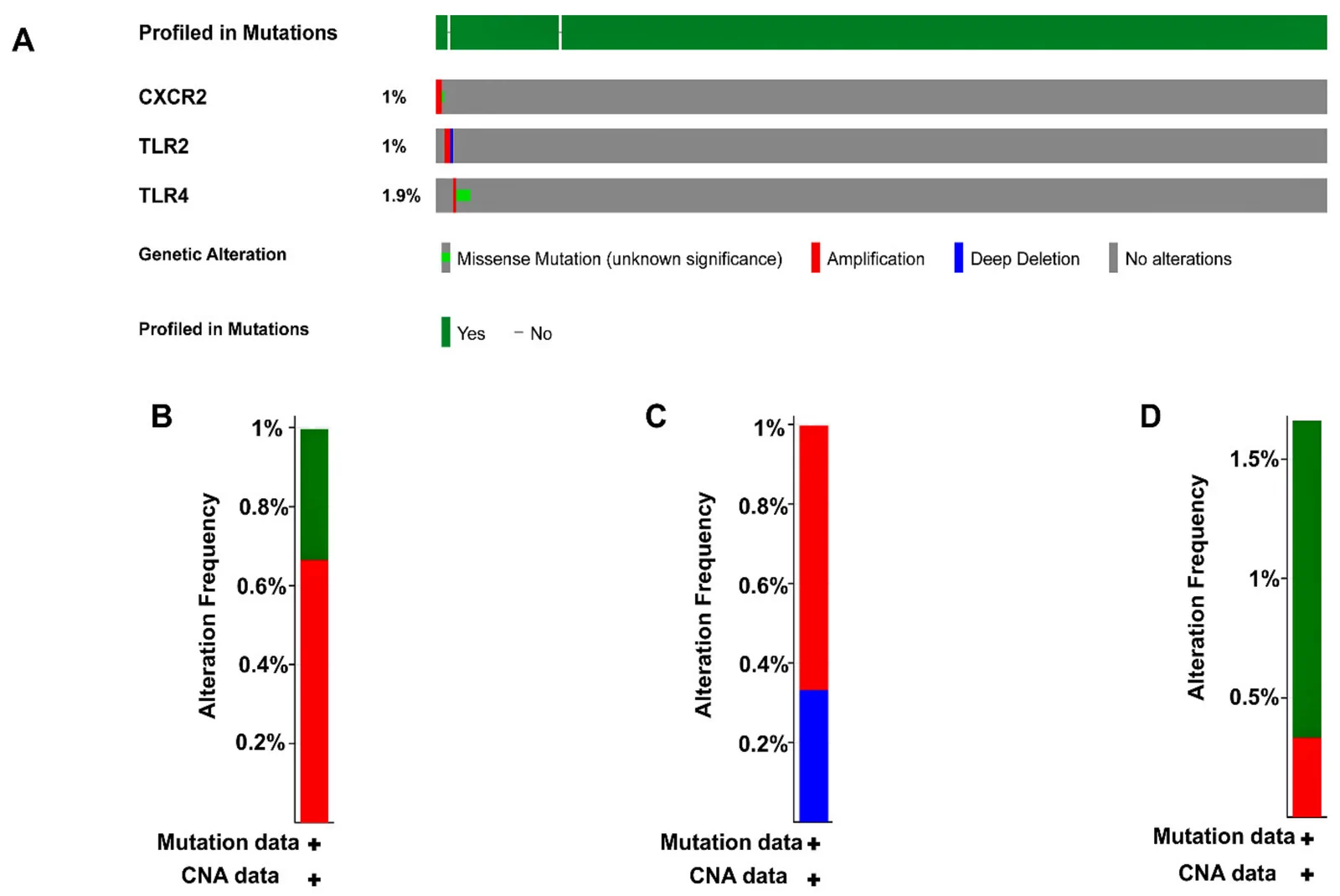
(**A**) The OncoPrint presents a concise overview of the genomic alterations in CXCR2, TLR2, and TLR4 within the TCGA-HCC cohort, consisting of 312 patient samples. In the lower row, the frequency of genomic alterations in CXCR2, TLR2, and TLR4 is represented, with red, blue, green, and gray bars denoting amplifications, deep deletions, missense mutations, and truncating mutations, respectively. Barplots in (**B**–**D**) display the frequencies of alterations in CXCR2, TLR2, and TLR4, respectively, across the 312 TCGA-HCC patient samples. In these plots, red, blue, and green bars signify amplifications, deep deletions, and missense mutations, respectively.

**Figure 6:**
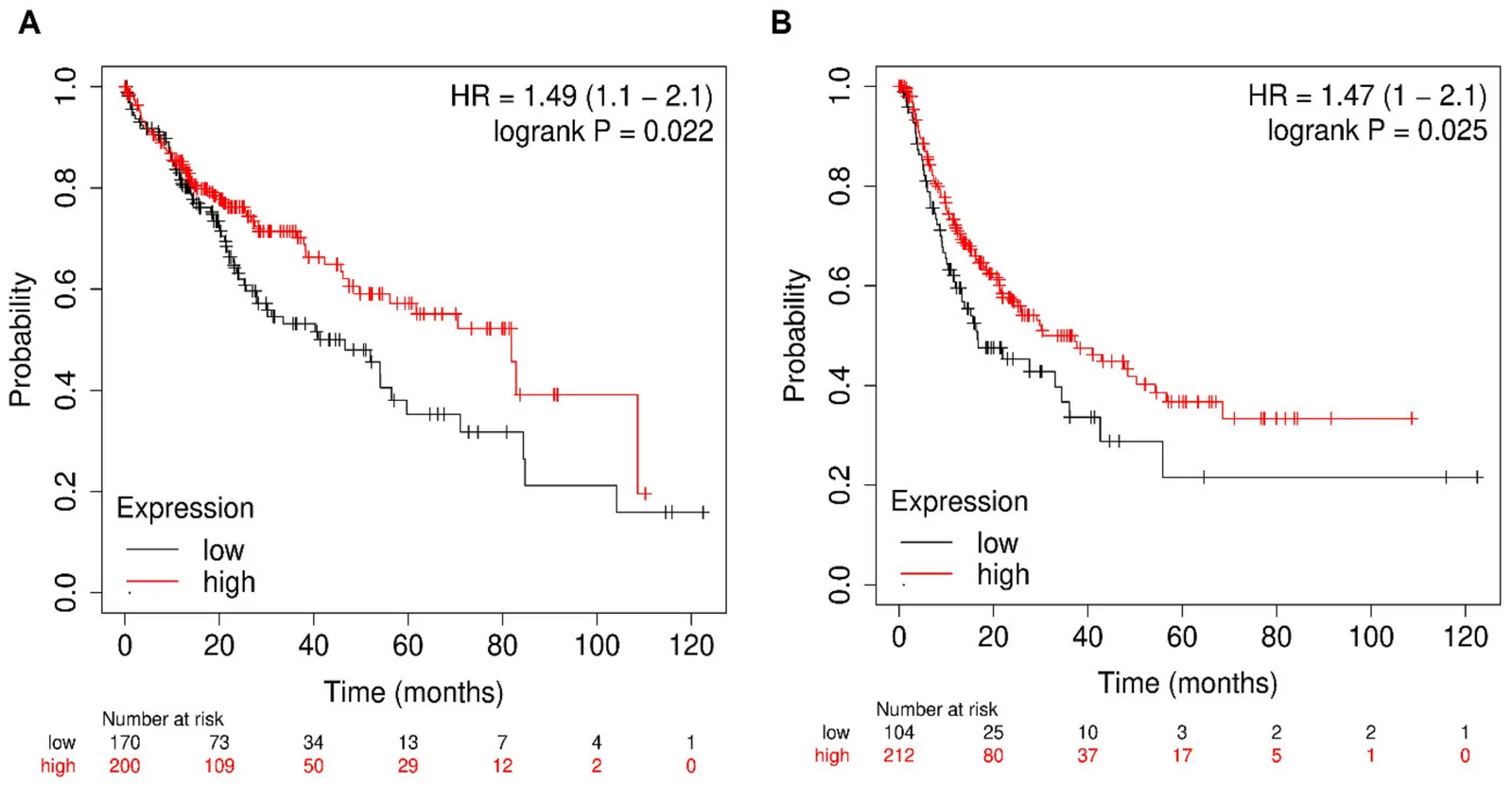
Kaplan–Meier (KM) plots showing (**A**) OS and (**B**) RFS of CXCR2 across 371 HCC patient samples. Lines colored in black and red correspond to HCC samples with lower and higher expression levels, respectively. Decreased expression of CXCR2 is associated with lower OS and RFS in HCC patients.

Genomic alterations in *CXCR2*, *TLR2*, and *TLR4* were investigated across 312 tumor samples of the TCGA-HCC cohort. The OncoPrint in Figure 5A reveals that *CXCR2*, *TLR2*, and *TLR4* were altered in 4% (12/312) of patient samples. *CXCR2*, *TLR2*, and *TLR4* reported 1%, 1%, and 1.9% mutation frequencies. The barplots shown in Figure 5B–D represent the overall alteration frequencies of *CXCR2*, *TLR2*, and *TLR4* based on the cancer-type summary analysis. Further, 0.66% (2/312 samples), 0.66% (2/312 samples), 0.33% (1/312 samples) amplification frequencies were observed in the case of *CXCR2*, *TLR2*, and *TLR4*. Additionally, 0.33% (1/312 samples) and 1.33% (4/312 samples) missense mutation frequencies were observed in the case of *CXCR2* and *TLR4*. Furthermore, 0.33% (1/312 samples) deep deletion frequency was observed in the case of *TLR2*. Only *CXCR2* reported significant differences between low- and high-expression cohorts for OS and recurrence-free survival (RFS) analyses. As shown in Figure 6A,B, KM plots revealed that poor OS and RFS were associated with lower mRNA expression levels of *CXCR2*. The hazard ratio (HR), 95% confidence interval (CI), and down- and up-expression cohort median survival time values of *CXCR2* w.r.t. OS and RFS are summarized in Table S10.

### 2.6. Validation Using TIMER and UALCAN

**Figure 7:**
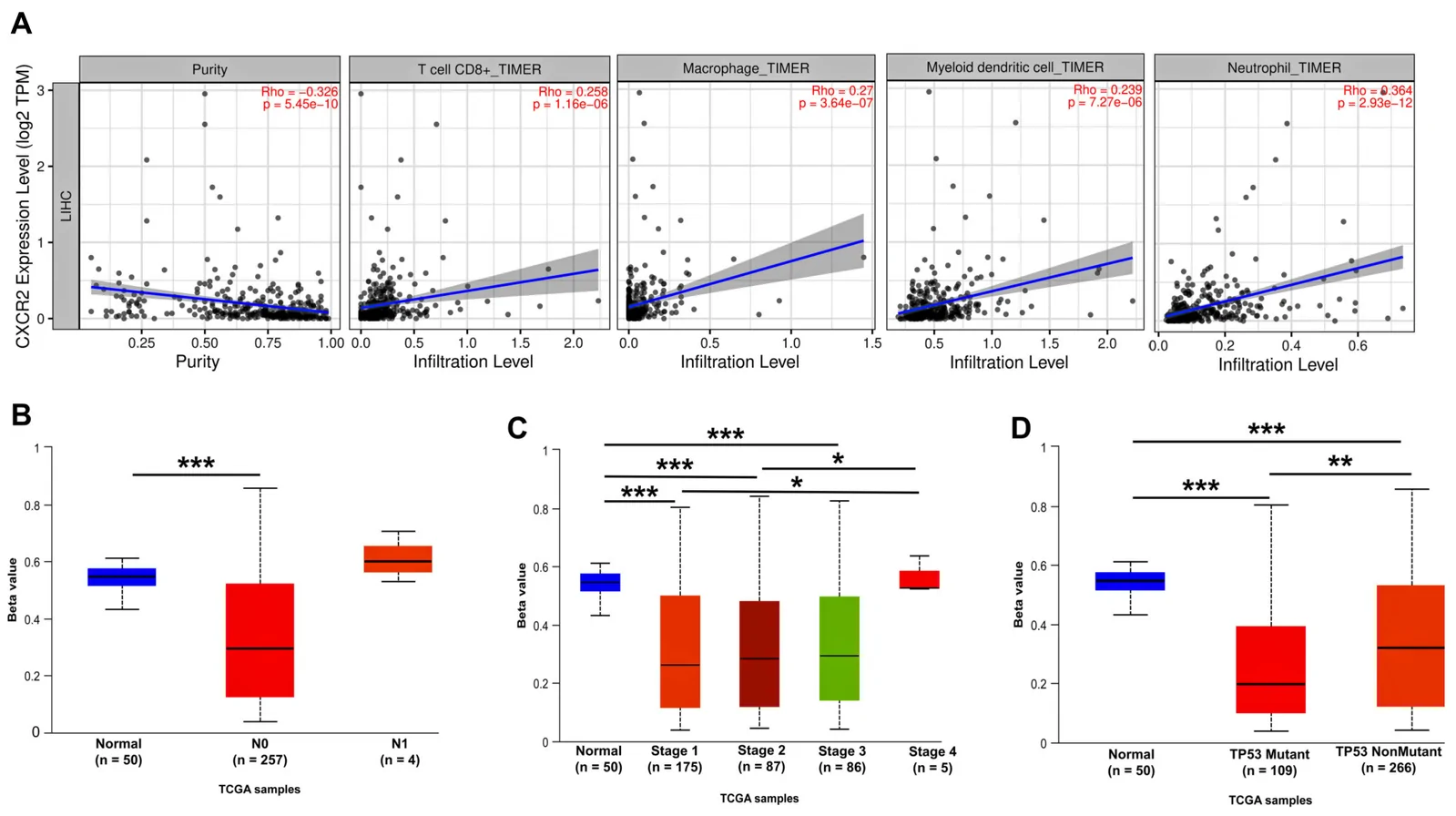
(**A**) The scatterplots reveal significant correlations between CXCR2 mRNA expression levels and the levels of infiltrating T cells, macrophages, DCs, and neutrophils within the TCGA-HCC cohort. Additionally, mRNA expression levels in relation to tumor purity are illustrated in the left panel of the figures. For each scatter plot, the Spearman’s correlation coefficient and the associated statistical significance are provided. (**B**) The box-and-whisker plots illustrate the distribution of CXCR2 promoter methylation based on normal and nodal metastasis (N0, N1) status. (**C**) Similarly, the box-and-whisker plots depict the distribution of CXCR2 promoter methylation based on normal and tumor stages (stage I, stage II, stage III, stage IV). (**D**) TP53 mutation status across the TCGA-HCC cohort. * *p*-value <0.05, ** *p*-value <0.01, *** *p*-value <0.001. The beta value (*y*-axis) indicates the level of DNA methylation ranging from 0 (unmethylated) to 1 (fully methylated). Different beta value cut-offs have been considered to indicate hypermethylation [Beta value: 0.7–0.5] or hypomethylation [beta value: 0.3–0.25].

The scatterplots in Figure 7A show the correlation of *CXCR2* mRNA expression levels with tumor purity and infiltrating levels of T cells, macrophages, dendritic cells (DCs), and neutrophils across the TCGA-HCC cohort. *CXCR2* had significant positive correlations with infiltrating levels of T cells (r=0.258, *p*-value $=1.16\times 10^{-6}$), macrophages (r=0.27, *p*-value $=3.64\times 10^{-7}$), DCs (r=0.239, *p*-value $=7.27\times 10^{-6}$), and neutrophils (r=0.364, *p*-value $=2.93\times 10^{-12}$). In addition, *CXCR2* showed a significant negative correlation with tumor purity (r=−0.326, *p*-value $=5.45\times 10^{-10}$) across the TCGA-HCC cohort. The box-and-whisker plots shown in Figure 7B–D reveal promoter methylation levels of *CXCR2* based on cancer stage, nodal metastasis, and TP53 mutation status. We observed a significant decrease in *CXCR2* from normal to N0 and thereafter a non-significant increase from N0 to N1 based on nodal metastasis promoter methylation analysis. In the case of the cancer stage promoter methylation analysis, we observed a significant decrease in *CXCR2* across stages I−III versus normal. Also, we observed a significant increase in *CXCR2* between stage I versus stage IV and stage II versus stage IV, respectively. In the case of TP53 mutation status, *CXCR2* was significantly decreased between normal versus TP53 mutant and normal versus TP53 non-mutant. *CXCR2* was slightly significantly elevated from the TP53 mutant to the TP53 non-mutant state.

## 3. Discussion

HCC’s increased mortality rate highlights the need for new biomarkers, therapeutic agents, and molecular therapeutic targets to be identified and discovered to further the development of early detection and efficient therapy. To create an effective therapeutic, it is crucial to identify gene targets related to the cancer’s characteristics. Currently, in silico methods are used on a bigger scale to identify the essential regulating genes [16,21,22,23]. The distinction and quantification of gene expression levels between normal and malignant samples is made possible through the analysis of gene expression profiles from several databases. Therefore, the primary goal of this suggested inquiry was to identify and validate important regulatory genes that are linked to HCC phenotypes using in silico methods. Understanding the patterns of variable gene expression in HCC may help develop novel diagnostic and therapeutic strategies and provide insights into the underlying molecular causes of the illness.

HCC is largely influenced by the immune system, which can both encourage tumor growth and act as a potential control mechanism [24]. HCC develops and progresses through several immune-related routes and processes. Our research also reports the maximum number of genes (Table S5) involved in immune system pathways. Chronic inflammation and viral infections, particularly hepatitis B and C, are major risk factors for HCC. Inflammatory pathways, driven by pro-inflammatory cytokines and chemokines, can contribute to the development and progression of HCC [25]. To strengthen the immune response (IR) to HCC and counteract the tumor’s immunosuppressive methods, research is being conducted to better understand the immune-related pathways in HCC and to create immunotherapies and combination treatments.

The inflammatory response is an important factor in initiating and spreading HCC [26]. Numerous inflammatory pathways and processes are implicated in the development of HCC, and chronic inflammation is a known risk factor for the disease. Our research reports a list of the top significant GO-BP terms corresponding to hub module DEGs (Table S6). Chronic liver illnesses, including liver cirrhosis, alcohol-related liver disease (ARLD), NAFLD, and chronic viral hepatitis (hepatitis B and C), are associated with high HCC cases. These disorders are typified by persistent inflammation of the liver, which over time may cause the liver to fill with fibrous scar tissue. Persistent inflammation in the liver can cause fibrosis, or the buildup of fibrous scar tissue, and ultimately cirrhosis [27]. Potential methods to lessen the risk and progression of HCC include targeting pro-inflammatory pathways, managing chronic liver disorders, and reducing inflammation. Furthermore, current studies and clinical trials center on the use of immunomodulatory and anti-inflammatory drugs, such as immune checkpoint inhibitors, in HCC therapy.

Presenting antigens to T cells and assisting in the coordination of the IR, MHC proteins, also referred to as human leukocyte antigen (HLA) proteins in humans, are essential components of the immune system [24]. MHC proteins play a critical role in the recognition and defense against foreign invaders, such as cancer cells. An essential component of the IR against cancer in the setting of HCC is the binding of MHC proteins to antigens [28]. Our results reported a list of top significant GO-MF terms, with MHC protein binding being most significant (Table S7). It is essential to comprehend how MHC protein binding affects the IR to HCC in order to design immunotherapies and individualized treatment plans. A possible strategy to strengthen the IR against HCC and boost treatment results is to take advantage of MHC–antigen interactions.

Membrane-bound vesicles called secretory granules are present in many types of cells, including some tumor cells. Cancer research is interested in the presence of secretory granules within tumor cells in the context of HCC [29]. Secretory granules are generally linked to the release and storage of a variety of chemicals, including neurotransmitters, hormones, and other bioactive compounds. But the importance of secretory granules in HCC varies, and their existence can be connected to a number of variables, including the tumor’s stage of differentiation and the particular proteins or substances kept in these granules. Our results report the most significant GO-CC terms, with secretory granule membrane comprising the maximum number of genes (Table S8). It is crucial to remember that the existence and importance of secretory granules in HCC might differ from case to case and may be influenced by the particular traits of the tumor. More investigation is required to completely understand the physiological significance of secretory granules in HCC and their potential as indicators for diagnosis or treatment.

*TLR2* is a member of the Toll-like receptor family, which plays a crucial role in the innate immune system’s recognition of microbial pathogens [30]. *TLR2* is expressed on the surface of various immune cells, including macrophages, DCs, and neutrophils, as well as on some non-immune cells [31]. In the context of HCC progression, *TLR2* has been implicated in several ways. Persistent inflammation is a hallmark of chronic liver diseases, such as hepatitis B and C infections and ARLD, which are known risk factors for HCC. Chronic activation of *TLR2* in the liver due to infections or other factors can contribute to prolonged inflammation. This chronic inflammation can promote fibrosis, cirrhosis, and HCC development over time. *TLR2* is expressed on immune cells and can also be found on HCC cells themselves. When *TLR2* is activated on tumor cells, it can produce pro-inflammatory chemokines, cytokines, and growth factors (GFs). This can create a tumor microenvironment (TME) that supports tumor growth, angiogenesis (formation of new blood vessels), and invasion. *TLR2* activation in the TME can also promote immunosuppression. It can lead to the recruitment of Tregs and myeloid-derived suppressor cells (MDSCs), which suppress the anti-tumor IR. This immune evasion strategy can allow HCC to progress unchecked [32].

In the context of HCC progression, *TLR4* has been implicated in several ways. *TLR4* is primarily known for its role in recognizing lipopolysaccharide (LPS) [33], a component found in the cell walls of Gram-negative bacteria (GNB). Activation of *TLR4* by LPS can lead to the initiation of an inflammatory response [34]. Chronic activation of *TLR4* in the liver due to bacterial infections or other factors can contribute to prolonged inflammation. This chronic inflammation is a key driver of liver fibrosis, cirrhosis, and ultimately HCC development. *TLR4* is expressed on various immune cells, including macrophages and DCs, as well as on some non-immune cells. Activation of *TLR4* on these cells can produce pro-inflammatory cytokines, chemokines, and GFs [35].

The innate immune systems of both *TLR2* and *TLR4* play key roles in the recognition and response to pathogens, such as bacteria and viruses [36]. These receptors are significant in the setting of HCC, as chronic inflammation, frequently linked to infections and IRs, is an important element in the onset and development of HCC [37]. Chronic liver diseases, such as hepatitis B and C infections, ARLD, and NAFLD, precede many cases of HCC. *TLR2* and *TLR4* are expressed on immune cells within the liver and can be activated by pathogen-associated molecular patterns (PAMPs), including components of bacteria and viruses [38]. Chronic activation of these receptors due to persistent infections or damage-associated molecular patterns (DAMPs) released from damaged liver cells can lead to prolonged inflammation in the liver, promoting fibrosis, cirrhosis, and ultimately HCC development. *TLR2* and *TLR4* activation triggers an IR, releasing pro-inflammatory cytokines and chemokines [39]. This can result in the recruitment of immune cells to the liver, including macrophages and neutrophils. In the context of chronic liver inflammation, this IR can contribute to tissue damage and the development of HCC. The interaction between *TLR2*, *TLR4*, and HCC is nuanced and varies based on the etiology of the liver disease, the stage of the tumor, and the immunological milieu. The roles of *TLR2* and *TLR4* in HCC are still being studied, as well as their potential as therapeutic targets for this malignancy.

*CXCR2* is a cell surface receptor that plays a role in the development and progression of various cancers, including HCC [40]. *CXCR2* is primarily known for its involvement in inflammation and the IR, as it binds to chemokines such as *IL8* or *CXCL8* and other related ligands [41]. *CXCR2* signaling has been linked to the promotion of tumor growth and metastasis in HCC [42]. Activation of *CXCR2* by its ligands can lead to increased tumor cell proliferation, angiogenesis (formation of new blood vessels to supply nutrients to the tumor), and invasion into surrounding tissues [43]. This contributes to the aggressive nature of HCC. *CXCR2* is involved in recruiting immune cells, such as neutrophils and tumor-associated macrophages (TAMs), to the TME in breast cancer [44]. While immune cells are essential for mounting an anti-tumor response, in HCC, their presence can also have pro-tumor effects. TAMs, for example, can promote tumor growth and angiogenesis [42]. Chronic inflammation is a known risk factor for the development of HCC, especially in the context of underlying liver diseases like hepatitis B or C and cirrhosis. *CXCR2*-mediated signaling can exacerbate inflammation, which, in turn, can contribute to the progression of liver disease and increase the risk of HCC. Because of its role in promoting tumor growth and metastasis, *CXCR2* has been considered a potential therapeutic target in HCC [45]. Inhibitors or antagonists of CXCR2 signaling that suppress HCC progression and improve treatment outcomes are being investigated in preclinical and clinical studies.

An important consideration when interpreting the present findings is that the analyses were performed using bulk RNA-sequencing data, in which measured transcript abundance represents the combined contribution of malignant cells and multiple stromal and immune cell populations. The observed positive correlation between CXCR2 expression and immune cell infiltration, together with its negative correlation with tumor purity, suggests that bulk CXCR2 expression may partially reflect the composition of the TME. Therefore, although our findings demonstrate that CXCR2 is significantly associated with prognosis and immune infiltration in HCC, they should not be interpreted as definitive evidence of a tumor cell-intrinsic role of CXCR2. Instead, CXCR2 should be regarded as a biomarker associated with the immune landscape of HCC whose mechanistic contribution requires confirmation using cell type-specific approaches such as single-cell RNA sequencing, spatial transcriptomics, immunohistochemistry (IHC), and multivariable analyses accounting for tumor purity and immune cell abundance.

In addition to transcriptomic discovery, we employed a multi-layered validation strategy to improve the robustness of our findings. The identified hub genes were first evaluated in an independent GEO cohort to assess the reproducibility of their differential expression. Furthermore, prognostic significance was investigated using KM survival analysis, which identified CXCR2 as the only hub gene significantly associated with both OS and RFS. To further evaluate the biological relevance of CXCR2, TIMER analysis demonstrated significant associations with tumor purity and immune cell infiltration, while UALCAN analysis provided complementary evidence across different clinicopathological subgroups. Collectively, these orthogonal validation approaches strengthen the evidence supporting CXCR2 as a clinically relevant biomarker beyond simple differential-expression overlap. Nevertheless, we acknowledge that module-preservation analysis and survival validation in additional independent HCC cohorts would provide further confirmation of the robustness of the identified co-expression network and represent important directions for future investigation.

## 4. Materials and Methods

### 4.1. HCC-Associated RNA-Seq Data Extraction and DEA

UCSC Xena browser [46] (https://xenabrowser.net/, accessed on 1 February 2026) was accessed in order to retrieve mRNA HTSeq-count data pertaining to the TCGA-HCC cohort. We picked patients in accordance with the following inclusion criteria: (1) there must be availability of count data from both tumor and normal patient samples; (2) there must be availability of definitive TNM classification information of tumor samples; (3) a proper record of gender, age, and race corresponding to samples should be available. To maintain uniformity, these samples were further cross-checked with mRNA-seq samples within the TCGA-GDC data portal. Solid tissue normal and solid tumor (primary) samples were retained in the cohort, followed by back-log-transforming the originally downloaded count file to obtain integer count values. Data normalization and ${log}_{2}$ transformation was attained via variance-stabilizing transformation (VST) of the back-log-transformed data using the DESeq2 v1.52.0 R package [47]. Sequential steps of batch correction, gene mapping, and duplicate gene handling were performed as discussed previously [20]. Genes with approved HUGO Gene Nomenclature Committee (HGNC) symbols were cross-checked and retained from the HGNC multi-symbol checker (https://www.genenames.org/tools/multi-symbol-checker/, accessed on 1 February 2026). A two-sample unpaired *t*-test via limma [48] was applied on all genes to screen DEGs at a statistical threshold of *p*-value <0.05 and $\left|{log}_{2}(fold\;change)\right|>0.2$. The cutoff was chosen after assessing all possible cutoffs, as at this particular threshold there was maximum coverage of DEGs for further downstream analysis and increasing the stringency would result in zero or a handful of DEGs.

### 4.2. HCC-Associated WGCN Formation and Hub Module Selection

Before establishing WGCN, we used the Pigengene v1.38.0 R package [49] to eliminate any noisy HCC-associated DEGs. Also, we removed any sample outliers during the quality check (QC) step as discussed previously [50]. We proceeded with the computation of the appropriate value in accordance with SFT criteria via the WGCNA v1.74 R package [51]. Steps of WGCN formation and module(s) selection were performed sequentially as discussed previously [50,52,53,54]. Since we were interested in identifying trait-linked modules, the age information of all subjects was also considered while computing ME and MEdiss. Next, we calculated the WGCN module topological measures such as MM, GS, and k.in to select the hub module(s). Module(s) with the highest correlations between MM versus k.in, MM versus GS, and GS versus k.in were considered as our hub module(s).

### 4.3. Functional Enrichment and PPIN Modular Analyses

DEGs present within the hub module(s) were submitted to Enrichr [55,56] (https://maayanlab.cloud/Enrichr/, accessed on 25 February 2026) and STRING v12.0 [57] (https://string-db.org/, accessed on 25 February 2026) databases. Reactome, GO-BP, GO-MF, GO-CC libraries were utilized for compilation of the top 10 significant (BH-*p*-value <0.05) data points corresponding to pathway and GO terms, respectively. Parameters used for PPIN establishment and cluster selection via molecular complex detection (MCODE) have been discussed previously [58]. DEGs overlapping between the top-scoring PPIN cluster and significant pathway/GO terms data were considered hub DEG(s).

### 4.4. Validation of Hub DEGs via GEO

“HCC” and “Hepatocellular carcinoma” were utilized as suitable keywords for searching the National Center for Biotechnology Information (NCBI)-GEO [59] (https://www.ncbi.nlm.nih.gov/geo/, accessed on 13 March 2026) exhaustively in order to fetch HCC-linked mRNA expression profiles. The exclusion and inclusion criteria for appropriate dataset selection have been discussed previously [58,60]. A sequential protocol of batch correction, gene mapping, and duplicate removal was performed as discussed previously [61]. DEGs were filtered via limma corresponding to a BH-*p*-value  < 0.01 and $\left|{log}_{2}(fold\;change)\right|>0.5$. Lastly, the presence of hub DEGs was cross-checked in the GEO DEGs list.

### 4.5. Mutational and OS Analyses

We accessed the cBioPortal [62,63] (https://www.cbioportal.org/, accessed on 15 March 2026) database to explore the genomic alterations in hub DEG(s). We selected the HCC (TCGA, Firehose Legacy) cohort in cBioPortal and chose all those tumor samples initially used for DEA. KM plots showing the OS and RFS of hub DEG(s) were displayed via the KM plotter database [64] (https://kmplot.com/analysis/, accessed on 19 March 2026) corresponding to TCGA-HCC cohort patient samples as per parameters discussed previously [2].

### 4.6. Validation Using TIMER and UALCAN

The TIMER web-based tool [65] (http://timer.cistrome.org/, accessed on 22 March 2026) was queried to discover the correlation between prognostically significant hub DEG(s) and T cells, macrophages, DCs, and neutrophils. A *p*-value <0.05 was regarded as the statistically significant threshold, along with Spearman’s test which was used for computing the correlation coefficient. Thereafter, we queried the UALCAN web-based tool [66,67] (http://ualcan.path.uab.edu/, accessed on 24 March 2026) for investigating the promoter methylation level of prognostically significant hub DEG(s) based on cancer stages, nodal metastasis, and TP53 mutation status.

## 5. Conclusions

In conclusion, this integrative bioinformatics study identified CXCR2 as a candidate hub gene associated with HCC through differential expression, WGCNA, PPIN analysis, functional enrichment, external transcriptomic validation, survival analysis, immune infiltration analysis, and promoter methylation assessment. The observed associations suggest that CXCR2 is closely linked to the HCC TME and patient prognosis. However, the present findings are derived from bulk transcriptomic analyses, and therefore the observed correlations between CXCR2 expression, immune cell infiltration, and tumor purity indicate that its expression may partially reflect the cellular composition of the TME rather than exclusively malignant hepatocytes. Consequently, the present study identifies CXCR2 as a candidate prognostic biomarker and a potential therapeutic candidate that warrants further investigation, rather than as a validated diagnostic, predictive, or therapeutic biomarker. Future studies incorporating independent clinical cohorts, multivariable survival analyses, single-cell and spatial transcriptomic approaches, and functional experimental validation will be essential to define the cell-type-specific role, biological mechanisms, and clinical utility of CXCR2 in HCC.

## Data Availability

The original contributions presented in this study are included in the article and Supplementary Materials. Further inquiries can be directed to the corresponding author.

## Supplementary Materials

The following supporting information can be downloaded at: https://www.mdpi.com/article/10.3390/ijms27167501/s1.
