# Supplementary material for: Decoding Tumor–Immune Interactions in Hepatocellular Carcinoma Through Network-Centered Identification of CXCR2
Source: Int J Mol Sci. 2026 Aug 21;27(16):7501. doi: 10.3390/ijms27167501 (PMC13513473; doi:10.3390/ijms27167501)
Supplement: Supplementary file 1 [file ijms-27-07501-s001.zip › SUPPLEMENTARY-1.pdf]

Supplementary Information

Table S1. *Clinical patients' information from TCGA-HCC cohort.*

Characteristics	Samples (n = 360)	Tumor (n = 315)	Normal (n = 45)
**Age**			
< 65	214	196	18
≥ 65	146	119	27
**Race**			
American Indian	01	01	–
Asian	156	150	06
White	186	152	34
Black	17	12	05
**Stage**			
Stage I	157	157	–
Stage II	81	81	–
Stage III	73	73	–
Stage IV	04	04	–
NT	45	–	45
**Gender**			
Male	242	217	25
Female	118	98	20

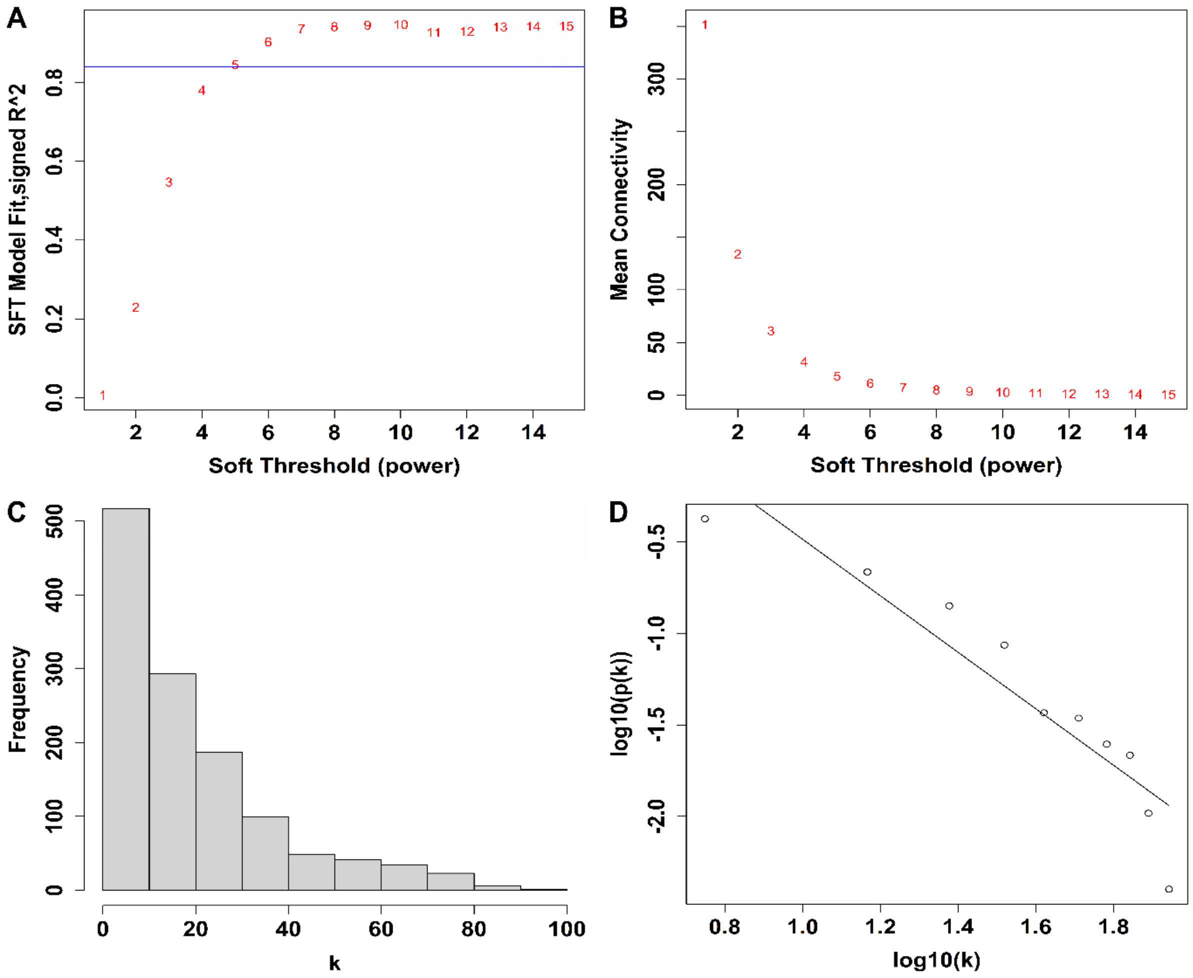

Figure S1. ***(A)*** *Analysis of scale-free fitting indices* ($\mathbf{R^2}$) *for various possible* $\boldsymbol{\beta}$ *values.* ***(B)*** *Analysis of mean connectivity for various possible* $\boldsymbol{\beta}$ *values.* ***(C)*** *Histogram of network connectivity distribution when* $\boldsymbol{\beta} = \mathbf{5}$. [*D*] $\boldsymbol{log_{10}(k)}$ *vs* $\boldsymbol{log_{10}(p(k))}$ *plot of the same histogram where the SFT is depicted by the approximate straight-line relationship (high* $\boldsymbol{R^2} = \mathbf{0.84}$*) and a negative value of slope* ($\boldsymbol{slope} = -\mathbf{1.57}$)*.*

Table S2. *MM vs k.in correlation and p-values for HCC-associated modules.*

Module	No. of genes	MM vs k.in (correlation)	MM vs k.in (p-value)
Blue	316	0.24	1.6×10^{-5}
Turquoise	452	0.99	$< 1 \times 10^{-200}$
Green	78	0.91	8.7×10^{-31}
Brown	267	0.97	8.7×10^{-165}
Yellow	137	0.72	3.6×10^{-23}

Table S3. *MM vs GS correlation and p-values for HCC-associated modules.*

Module	No. of genes	MM vs GS (correlation)	MM vs GS (p-value)
Blue	316	-0.093	9.9×10^{-2}
Turquoise	452	0.23	7.7×10^{-7}
Green	78	0.34	2.3×10^{-3}
Brown	267	-0.083	0.18
Yellow	137	0.29	5.9×10^{-4}

Table S4. *GS vs k.in correlation and p-values for HCC-associated modules.*

Module	No. of genes	GS vs k.in (correlation)	GS vs k.in (p-value)
Blue	316	-0.067	0.23
Turquoise	452	-0.13	6.0×10^{-3}
Green	78	0.054	0.6
Brown	267	-0.13	3.6×10^{-2}
Yellow	137	-0.29	7.4×10^{-4}

Table S5. *List of top* 10 *significant pathways corresponding to hub module DEGs.*

Pathway	Gene count	BH-p-value
Immune system	150	4.01×10^{-41}
Innate immune system	87	1.89×10^{-24}
Immunoregulatory interactions between a lymphoid and a non-lymphoid cell	33	5.61×10^{-24}
Neutrophil degranulation	56	1.72×10^{-22}
Adaptive immune system	64	1.51×10^{-18}
Signaling by GPCR	58	5.51×10^{-16}
GPCR downstream signaling	52	3.21×10^{-14}
Interleukin-10 signaling	15	2.87×10^{-12}
GPCR ligand binding	40	2.38×10^{-11}
Class A/1 (rhodopsin-like receptors)	33	5.44×10^{-11}

Table S6. *List of top* 10 *significant GO-BP terms corresponding to hub module DEGs.*

GO-BP	Gene count	BH-p-value
Inflammatory response (GO:0006954)	49	1.27×10^{-29}
Positive regulation of cytokine production (GO:0001819)	46	9.23×10^{-21}
Negative regulation of T cell activation (GO:0050868)	19	2.91×10^{-16}
Negative regulation of cytokine production (GO:0001818)	30	4.05×10^{-15}
Antigen receptor-mediated signaling pathway (GO:0050851)	26	1.46×10^{-14}
Regulation of T cell proliferation (GO:0042129)	18	1.40×10^{-13}
Negative regulation of lymphocyte proliferation (GO:0050672)	13	8.37×10^{-13}
Cellular defense response (GO:0006968)	14	1.22×10^{-12}
Positive regulation of lymphocyte proliferation (GO:0050671)	16	1.00×10^{-11}
T Cell Activation (GO:0042110)	19	1.22×10^{-11}

Table S7. *List of top* 10 *significant GO-MF terms corresponding to hub module DEGs.*

GO-MF	Gene count	BH-p-value
MHC protein binding (GO:0042287)	09	1.75×10^{-5}
Amyloid-beta binding (GO:0001540)	12	3.56×10^{-5}
G protein-coupled receptor activity (GO:0004930)	21	3.56×10^{-5}
Cytokine activity (GO:0005125)	17	5.22×10^{-5}
Inhibitory MHC class I receptor activity (GO:0032396)	05	5.22×10^{-5}
Cytokine receptor activity (GO:0004896)	11	6.85×10^{-5}
MHC class I receptor activity (GO:0032393)	05	1.15×10^{-4}
Complement receptor activity (GO:0004875)	05	1.15×10^{-4}
MHC class I protein binding (GO:0042288)	05	2.75×10^{-4}
Chemokine receptor binding (GO:0042379)	08	5.78×10^{-4}

Table S8. *List of top* 10 *significant GO-CC terms corresponding to hub module DEGs.*

GO-CC	Gene count	BH-p-value
Secretory granule membrane (GO:0030667)	42	2.20×10^{-20}
Tertiary granule membrane (GO:0070821)	22	2.07×10^{-17}
Tertiary granule (GO:0070820)	28	2.63×10^{-15}
Specific granule membrane (GO:0035579)	14	5.17×10^{-11}
Specific granule (GO:0042581)	19	5.17×10^{-11}
Ficolin-1-rich granule membrane (GO:0101003)	09	1.95×10^{-8}
Ficolin-1-rich granule (GO:0101002)	15	1.01×10^{-6}
Cytoplasmic vesicle membrane (GO:0030659)	24	1.73×10^{-6}
Membrane raft (GO:0045121)	13	5.95×10^{-6}
Plasma membrane raft (GO:0044853)	06	2.77×10^{-4}

Table S10. *HR,* $95\%CI$*, low and high expression median survival times of CXCR2 w.r.t. both survival types.*

Survival Type	HR	95%CI	Low expression cohort (months)	High expression cohort (months)
OS	1.49	$1.1 - 2.1$	46.57	81.87
RFS	1.47	$1 - 2.1$	16.6	30.4
